# Investigation of possible phase transition of the frustrated spin-1/2 *J*_1_-*J*_2_-*J*_3_ model on the square lattice

**DOI:** 10.1038/s41598-017-10986-8

**Published:** 2017-09-05

**Authors:** Ai-Yuan Hu, Huai-Yu Wang

**Affiliations:** 1 College of Physics and Electronic Engineering, Chongqing Normal University, Chongqing, 401331 China; 2 Department of Physics, Tsinghua University, Beijing, 100084 China

## Abstract

The frustrated spin-1/2 *J*_1_-*J*_2_-*J*_3_ antiferromagnet with exchange anisotropy on the two-dimensional square lattice is investigated. The exchange anisotropy is presented by *η* with 0 ≤ *η* < 1. The effects of the *J*_1_, *J*_2_, *J*_3_ and anisotropy on the possible phase transition of the Néel state and collinear state are studied comprehensively. Our results indicate that for *J*_3_ > 0 there are upper limits ${J}_{3}^{c}$ and *η* ^*c*^ values. When 0 < *J*_3_ ≤ ${J}_{3}^{c}$ and 0 ≤ *η* ≤ *η* ^*c*^, the Néel and collinear states have the same order-disorder transition point at *J*_2_ = *J*_1_/2. Nevertheless, when the *J*_3_ and *η* values beyond the upper limits, it is a paramagnetic phase at *J*_2_ = *J*_1_/2. For *J*_3_ < 0, in the case of 0 ≤ *η* < 1, the two states always have the same critical temperature as long as *J*_2_ = *J*_1_/2. Therefore, for *J*_2_ = *J*_1_/2, under such parameters, a first-order phase transition between the two states for these two cases below the critical temperatures may occur. When *J*_2_ ≠ *J*_1_/2, the Néel and collinear states may also exist, while they have different critical temperatures. When *J*_2_ > *J*_1_/2, a first-order phase transition between the two states may also occur. However, for *J*_2_ < *J*_1_/2, the Néel state is always more stable than the collinear state.

## Introduction

In recent years, the study of frustration quantum spin systems has become very active on regular two-dimensional (2D) lattices, for examples, square lattice^1–15^, triangular lattice^16–19^, honeycomb lattice^20–22^, kagome lattice^23–25^ and so on. One of the most intensively studied frustrated 2D models is the spin-1/2 *J*_1_-*J*_2_ Heisenberg antiferroamgnet on the square lattice where the nearest-neighbor (NN) bonds (of strength *J*_1_ > 0) competes with next-nearest-neighbor (NNN) bonds (of strength *J*_2_ = *αJ*_1_ > 0)^2–12^. This model has been widely investigated focused on its ground states by means of various theoretical methods^1–12^, such as the effective field theory^2^, cluster mean field theory^3^, density matrix renormalization group^4, 5^, exact diagonalization^5, 6^, bond-operator theory^8^, spin-wave theory^9, 10^, the coupled cluster method^11, 12^ and so on. The *J*_1_ - *J*_2_ model on the two-dimensional square lattices usually shows two possible antiferromagnetic states. One is called Néel state or AF1 state, and the other is called collinear state or AF2 state. These investigations showed that the system was a Néel state at $\alpha \lesssim 0.38$ and a collinear state for $\alpha \gtrsim 0.6$. In the range of 0.38 < *α* < 0.6, the square lattice system was nonmagnetic^2–12^, and the triangular lattice system might be a spin-liquid state^19^.

The 2D *J*_1_-*J*_2_ may be used to describe the magnetic properties of some real materials. Examples are the undoped precursors to the high temperature superconducting cuprates for small *α* values^26^, VOMoO_4_ for intermediate *α* values^27^, and Li_2_ VOSiO _4_ for large *α* values^28^. Meanwhile, experimental results indicated that for Li_2_ VOSiO _4_ the value of *α* can be changed from low to high by applying high pressure^29^.

For finite temperature, there is no long-range order for isotropic two-dimensional model^30^. Usually, an anisotropy is considered. This is because an anisotropy, no matter how faint, would cause a long-range order at finite temperature. Based on this fact, J. R. Viana *et al*. studied the phase diagram of an exchange anisotropic *J*_1_-*J*_2_ model^2^. Their results indicated that between the paramagnetic and collinear phases the system underwent a first-order transition at low temperature and a second-order transition at high temperature. T. Roscilde *et al*. investigation showed that when an Ising type exchange anisotropy was induced, there would be Chandra-Coleman-Larkin transition and Berezinskii-Kosterlitz-Thouless transition^9^. Their results showed that the anisotropy could effectively tune the quantum fluctuation and frustration of the system. These investigations indicated that the anisotropy played an important role.

As a more complicated model, the next-next-nearest-neighbor (NNNN) exchange is added to the *J*_1_-*J*_2_ model, so as to form the *J*_1_-*J*_2_-*J*_3_ model^31^. It possesses more degrees of freedom to tune the quantum fluctuation and frustration of the system compared to the *J*_1_-*J*_2_ model. Experimental investigations indicated that the AF2 and various transition behaviors exist in most of the iron-based superconductors. It was thought that further-neighboring interactions might be available and played an important role in determining the magnetic properties^32^. For example, a nonzero coupling *J*_3_ between the NNNN was suggested to be important for the magnetic properties in iron chalcogenides such as FeTe^33^.

Theoretical studied indicated that the classical ground state of the *J*_1_-*J*_2_-*J*_3_ model allowed four ordered phases due to the competing interactions *J*_2_/*J*_1_ and *J*_3_/*J*_1_^34–36^, i.e., Néel, collinear and two helicoidal states that were depicted in Fig. 1 of ref. 36. The nature of the zero temperature quantum phases in selected regions *J*_1_, *J*_2_, and *J*_3_ had been also studied by some authors^37, 38^. For the *J*_1_-*J*_2_-*J*_3_ model, work has mainly been focused on its ground state properties^34–38^. Investigations concerning nonzero temperature have been comparatively much fewer. One work we can see was the phase diagram at *J*_2_ = 0 by Luca *et al*.^39^. Meanwhile, because of so many parameters, the properties of the *J*_1_-*J*_2_-*J*_3_ model have not been clearly known yet. A detailed investigation is still desired.

**Figure 1:**
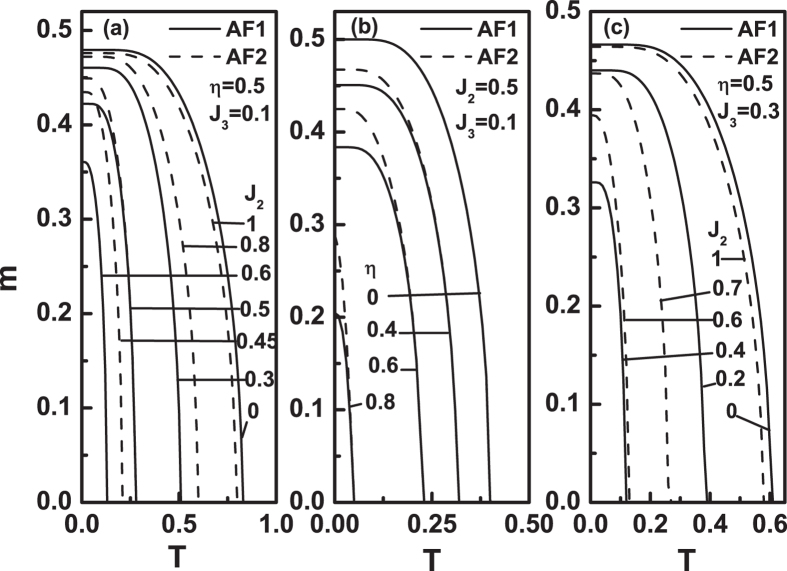
The sublattice magnetization *m* as a function of temperature *T* for different parameters. (**a**) *η* = 0.5, *J*_3_ = 0.1 and various *J*_2_ values. (**b**) *J*_2_ = 0.5, *J*_3_ = 0.1 and various *η* values. (**c**) *η* = 0.5, *J*_3_ = 0.3 and various *J*_2_ values.

In this paper, we comprehensively study the magnetic properties of the *J*_1_-*J*_2_-*J*_3_ model at finite temperature by using the double-time Green¡¯s function (DTGF) method. As mentioned above, an anisotropy is necessary at finite temperature. Here an exchange anisotropy *η* is considered. Note that the model is isotropic at *η* = 1. Thus we consider the cases when 0 ≤ *η* < 1. The *J*_1_ and *J*_2_ values are set to be positive and the *J*_3_ value can be either positive or negative. Our results show that, as *J*_3_ < 0 and *J*_2_ = *J*_1_/2, both AF1 and AF2 states can exist and have the same critical temperature in the whole anisotropy range 0 ≤ *η* < 1, but as *J*_3_ > 0, this conclusion holds merely in a part of the anisotropy range. When *J*_2_ ≠ *J*_1_/2, the two states may also exist, but their critical temperatures differ from each other. In this case, the calculated free energies show that a first-order phase transition between the Néel and collinear states below critical point may occur.

## Results

We discuss the properties at finite temperature. Therefore, when we say zero temperature, we actually mean that temperature is very close to zero, which is denoted by *T* = 0^+^.

### Basic magnetic properties

First, we discuss the case of *J*_3_ > 0. Figure 1 plots the magnetization *m* as a function of temperature *T* at various parameter values. In Fig. 1(a), *η* = 0.5 and *J*_3_ = 0.1. When *J*_2_ ≤ 0.45, it is AF1 configuration, and when *J*_2_ ≥ 0.6 the AF2 configuration. For the AF1 configuration, as *J*_2_ increases from zero, the competition between *J*_2_/*J*_1_ and *J*_3_/*J*_1_ emerges. Since we have fixed *J*_1_ = 1, this competition is actually between *J*_2_ and *J*_3_. For a fixed *J*_3_, the frustration becomes stronger with the increasing the value of *J*_2_. This leads to that *T*_*N*_ and *m* decrease with the increase of *J*_2_. For the AF2 configuration, this case is contrary, i.e., the frustration decreases with increasing of *J*_2_. As a result, both *T*_*N*_ and *m* increase with increasing *J*_2_ value. When 0.45 < *J*_2_ < 0.6, the system can be either AF1 or AF2 state, but with different *T*_*N*_ values except *J*_2_ = 0.5.

Now we focus on the curve of *J*_2_ ≤ 0.5 in Fig. 1(a) that both configurations can exist and have the same order-disorder transition point for *J*_3_ = 0.1 and *η* = 0.5. Let us see whether at *J*_2_ = 0.5 this conclusion is true for any other *J*_3_ and *η* values. We change *η* values and fix *J*_2_ = 0.5 and *J*_3_ = 0.1, and the results are shown in Fig. 1(b). It is seen that, as *η* takes value from 0 to 0.8, the critical temperatures of the two states are always equal. When *η* > 0.8, numerical calculation shows that the system is always a paramagnetic (P) phase. As *J*_3_ value increases, the system will be P phase at *J*_2_ = 0.5 even *η* is less than 0.8. Figure 1(c) shows $m \sim T$ curves for various *J*_2_ values when *η* = 0.5 and *J*_3_ = 0.3. When *J*_2_ ≤ 0.4, it is AF1 state and when *J*_2_ ≥ 0.6, it is AF2 state. Around *J*_2_ = 0.5, the system is a paramagnetic phase.

**Figure 2:**
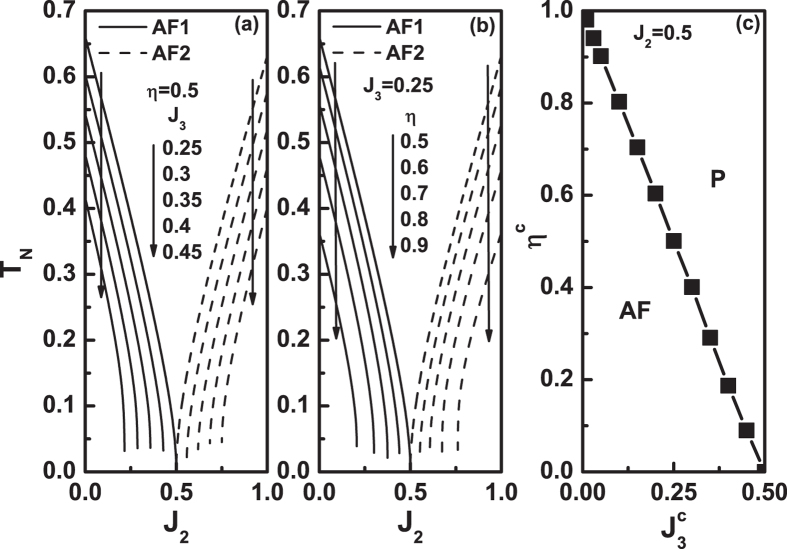
(**a**) and (**b**) The Néel temperature *T*_*N*_ as a function of *J*_2_ at different *J*_3_ and *η* values. (**a**) *η* = 0.5 and different *J*_3_ values. (**b**) *J*_3_ = 0.25 and different *η* values. (**c**) The relationship between *η* ^*c*^ and ${J}_{3}^{c}$ values when *J*_2_ = 0.5. This panel is divided into two regions, i.e., AF and P regions. The AF region representation is that AF1 and AF2 states have the same critical temperature at *J*_2_ = 0.5. For P region, it is a paramagnetic phase for AF1 and AF2 states at *J*_2_ = 0.5.

Since *T*_*N*_ value depends on the parameters, we have to know the details of the dependence. Figure 2(a),(b) plot *T*_*N*_ as a function of *J*_2_ for various *η* and *J*_3_ values. It is seen from Fig. 2(a) that, when *η* = 0.5 and *J*_2_ = 0.5, the two states have the same critical temperature at *J*_3_ = 0.25, although the *T*_*N*_ value is rather low. As *J*_3_ increases further, the system will be a P phase in the vicinity of *J*_2_ = 0.5, Fig. 1(c) showing one example. The range of *J*_2_ value where the system is P phase becomes larger with the increase of *J*_3_ value. It indicates that the increasing of the *J*_3_ value leads to a stronger frustration. In combination of Figs 2(a)and 1(a), it is drawn that for 0 ≤ *J*_3_ ≤ 0.25 the two states AF1 and AF2 have the same *T*_*N*_ value at *J*_2_ = 0.5. These results reflect that, for a fixed *η*, there is an upper limit *η* ^*c*^ below which the order-disorder points of the two states are the same. Note that the ${J}_{3}^{c}$ value will change with the change of *η*.

In Fig. 2(b), *J*_3_ = 0.25 is fixed and the *η* varies. Similar to Fig. 2(a), for a fixed *J*_3_, there is an upper limit *η* ^*c*^ value below which the two states have the same critical temperature at *J*_2_ = 0.5. Figure 2(c) shows the relation between the *η* ^*c*^ and ${J}_{3}^{c}$ values. It is a straight line and can be expressed by ${\eta }^{c}=-2{J}_{3}^{c}+1$. This line divides the panel into two regions, i.e., antiferromagnetic (AF) and P regions. In AF region, under the same *J*_1_, *J*_2_, *J*_3_ and *η* values, the two states have the same critical temperature. One example is the case of *J*_3_ = 0.25 and *η* = 0.5 shown in Fig. 2(a,b). In P region, the system is always a P phase. The examples are the curves with *J*_3_ ≥ 0.3 in Fig. 2(a) and that with 0.6 ≤ *η* < 1 in Fig. 2(b).

**Figure 3:**
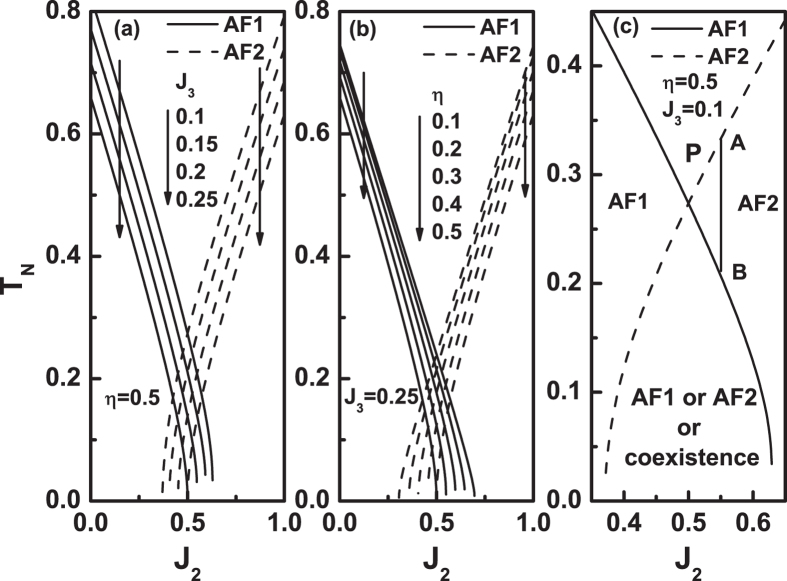
The Néel temperature *T*_*N*_ of AF region (see Fig. 2(c)) as a function of *J*_2_ at different *J*_3_ and *η* values. (**a**) *η* = 0.5 and various *J*_3_ values. (**b**) *J*_3_ = 0.25 and various *η* values. (**c**) The enlargement of the region of *J*_2_ in the vicinity of *J*_2_ = 0.5 when *η* = 0.5 and *J*_3_ = 0.1.

Figure 3 plots the critical temperature as a function of *J*_2_ for different *J*_3_ and *η* values. These panels are also phase diagrams. It is seen from Fig. 3(a) that the critical temperature increases with decreasing *J*_3_. As mentioned above, the frustration increases with increasing *J*_3_. For a fixed *J*_3_, the critical temperature decreases with the increase of *η*, see Fig. 3(b). It can be easily understood that a stronger anisotropy suppresses the quantum fluctuation of the system so that raises the critical point. Figure 3(a,b) indicate that the smaller the *J*_3_ and *η* values, the weaker the frustration.

We plot in Fig. 3(c) a pair of lines with the same parameters. This in fact is a phase diagram. The two lines divide the figure into four regions. The upper region, marked by P, means that the system is in P state. The left and right regions are that the system is in AF1 and AF2 configurations, respectively. The lower region is where the AF1 and AF2 configurations can coexist. From Fig. 3(a,b), it is obvious that when the *J*_3_ and/or *η* values decrease, the pair of lines in Fig. 3(c), as well as their cross point, will move upwards, and the lower region will expand. On the contrary, as the *J*_3_ and/or *η* values increase, the pair of lines in Fig. 3(c), as well as their cross point, will move downwards, and eventually, the two lines will be apart, as shown in Fig. 2(a,b).

Figure 3(a,b) also explicitly show that, when *J*_3_ and *η* take values in AF region, the two states have the same critical temperature as long as *J*_2_ = 0.5. As the *J*_2_ value is not equal to 0.5, both configurations can exist in low temperature as in the lower region in Fig. 3(c), but have different *T*_*N*_ values, see, for example, the points A and B in Fig. 3(c).

Please note that the solid and dashed lines are not symmetric with respect to *J*_2_ = 0.5 in Fig. 3(c), although it seems so. In fact, the value of the *J*_2_ does not have upper limit and the dashed line can extend to larger *J*_2_ values. Similarly, in Figs 2(a) and 3(a), each pair of the solid and dashed lines with the same *η* value is not symmetric with respect to *J*_2_ = 0.5, and neither is each pair lines with the same *J*_2_ value in Figs 2(b) and 3(b).

**Figure 4:**
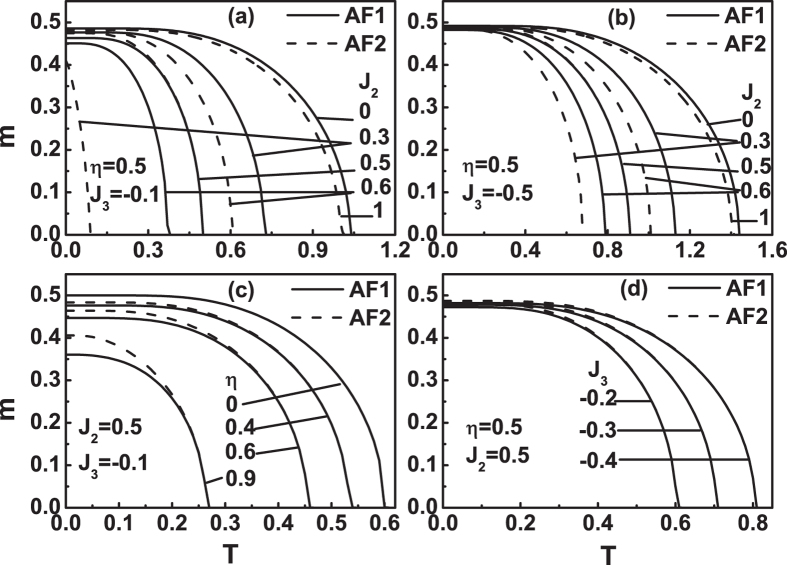
Sublattice magnetization as a function of temperature under various parameter values. (**a**) *η* = 0.5, *J*_3_ = −0.1 and *J*_2_ value varies. (**b**) *η* = 0.5, *J*_3_ = −0.5 and *J*_2_ value varies. (**c**) *J*_2_ = 0.5, *J*_3_ = − 0.1 and *η* value varies. (**d**) *η* = 0.5, *J*_2_ = 0.5 and *J*_3_ = −02, −0.3, −0.4.

Next, we discuss the case of *J*_3_ < 0. Figure 4 plots *m* as a function of *T* for various parameters. From Fig. 4(a,b), when *J*_2_ is near zero, it is AF1 state, and when *J*_2_ is near 1, the AF2 state. As the *J*_2_ is around 0.5, the state can be either AF1 or AF2. A remarkable feature for minus *J*_3_ value is that the AF1 and AF2 states always have the same critical temperature for 0 ≤ *η* < 1 as long as *J*_2_ = 0.5, which are explicitly shown in Fig. 4(c,d) by some examples. While for *J*_3_ > 0, only when the parameters are in the region AF in Fig. 2(c) can the two configurations have the same *T*_*N*_ values.

**Figure 5:**
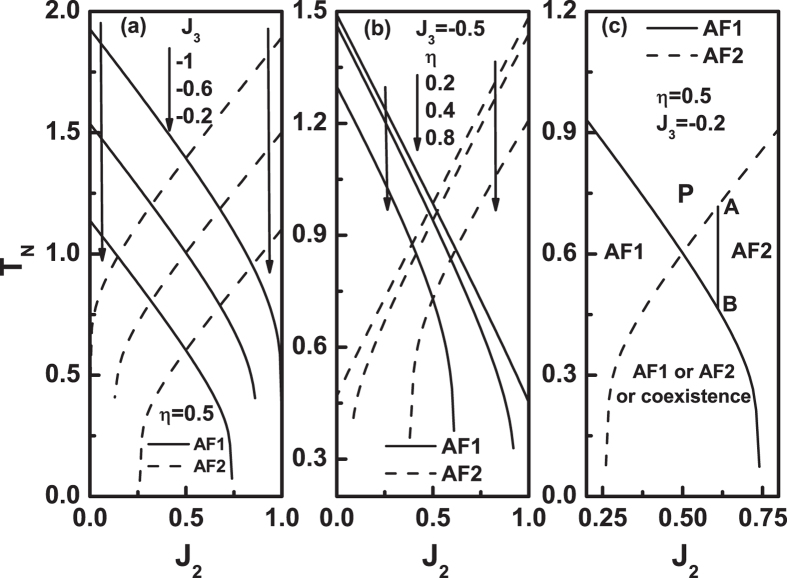
The Néel temperature *T*_*N*_ as a function of *J*_2_ at different *J*_3_ and *η* values. (**a**) *η* = 0.5 and *J*_3_ = − 0.2, −0.6, −1. (**b**) *J*_3_ = −0.5 and *η* = 0.2, 0.4, 0.8. (**c**) It is the case of *J*_3_ = −0.2 in (**a**).

Figure 5 illustrate the critical point *T*_*N*_ as a function of *J*_2_ value under different *J*_3_ and *η* parameters. Figure 5(a),(b) are similar to Fig. 2(a),(b) and Fig. 3(a),(b). However, a pair of lines with the same parameters in Fig. 5(a),(b) have always a cross point at *J*_2_ = 0.5. Figure 5(c) is similar to Fig. 3(c), and the discussion is either similar.

### Possible phase transition at *J*_2_ = *J*_1_/2

We have known from the discussion above that AF1 and AF2 may have the same critical temperature at 0 ≤ *η* < 1 when *J*_2_ = 0.5. A question naturally arises from this feature that which configuration is more stable at *J*_2_ = 0.5. In the following, we manage to answer this question. The two configurations are different from each other, and so are their entropies at a fixed temperature. Therefore, the internal energy cannot be used to determine which one is more stable at each temperature. Under the same volume and temperature, the state with lower free energy is more stable.

The free energy can be evaluated numerically by means of the internal energy via $F(T)=E\mathrm{(0)}-T$ ${\int }_{0}^{T}\frac{E(T^{\prime} )-E\mathrm{(0)}}{{T}^{\mathrm{\text{'}2}}}dT^{\prime}$, where *E*(*T*) represents the internal energy of the system, which is defined as the thermostatistical average of Hamiltonian, *E* = (<*H*>)/(*N*)^40^. Computing internal energy involves the calculation of the transverse ($\sum _{i,j} < {S}_{i}^{+}{S}_{j}^{-} >$) and longitudinal ($\sum _{i,j} < {S}_{i}^{z}{S}_{j}^{z} >$) correlation functions. We do not present the lengthy derivation. The formulism was presented in ref. 41.

In the following, the influence of *J*_3_ and *η* on the possible phase transition between AF1 and AF2 states are studied. In this section, we discuss the case of *J*_2_ = *J*_1_/2.

**Figure 6:**
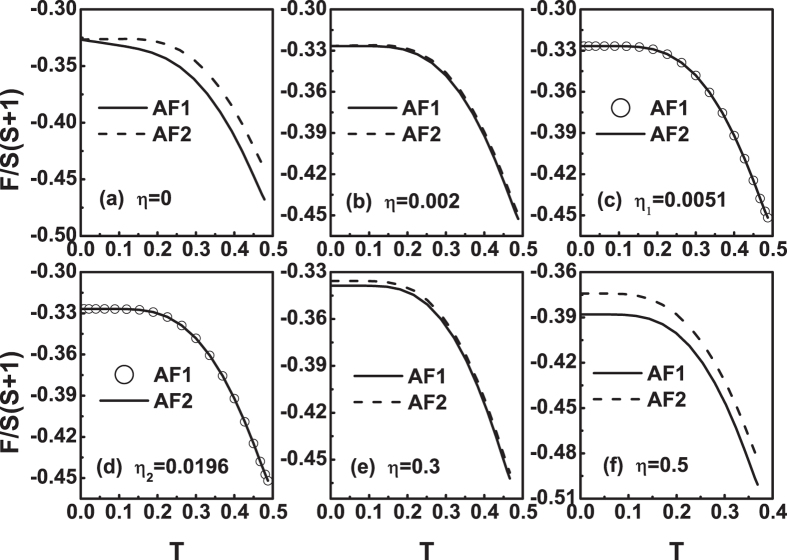
The free energy *F*(*T*) curves at *J*_3_ = 0.01 for different *η* values. (**a**) *η* = 0, (**b**) *η* = 0.002, (**c**) *η* = *η*_1_ = 0.0051, (**d**) *η* = *η*_2_ = 0.0196, (**e**) *η* = 0.3 and (**f**) *η* = 0.5.

Figure 6 plots the free energy as a function of temperature for different *η* values at *J*_3_ = 0.01. Figure 6(a) shows that at *η* = 0 the free energy of AF1 is always less than AF2 at finite temperature. When temperature close to zero, their free energies seem the same, but actually, *F*_*AF*1_(0^+^) < *F*_*AF*2_(0^+^). Therefore, in this case, AF1 is more stable in the range of *T* ≤ *T*_*N*_. As *η* increases from zero, the free energy curves of the two states become closer gradually, see Fig. 6(b). When *η* increases to *η*_1_ = 0.0051, the difference between the free energies of the AF1 and AF2 is negligible, see Fig. 6(c). This situation will last until *η*_2_ = 0.0196, see Fig. 6(d). This case means that at *J*_3_ = 0.01 the system can be in either the AF1 or AF2 state or the coexistence of them for *η*_1_ ≤ *η* ≤ *η*_2_. When *η* increases further from *η*_2_. The free energy curves of the two states begin to separate. Then, the *F*_*AF*1_(*T*) curve drops faster than *F*_*AF*2_(*T*) curve at *T* ≤ *T*_*N*_, see Fig. 6(e),(f). On the whole, as *η* increases from zero, the two *F*(*T*) curves moves downwards.

**Figure 7:**
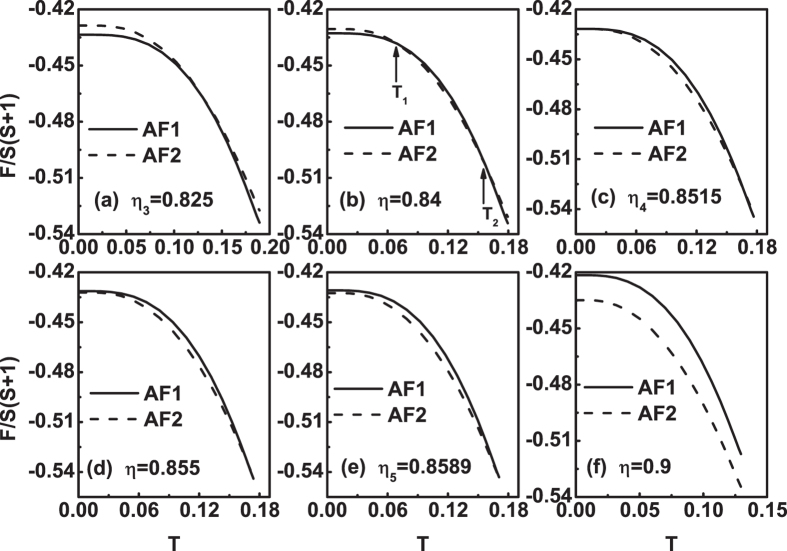
The free energy *F*(*T*) curves at *J*_3_ = 0.01 for different *η* values. (**a**) *η*_3_ = 0.825, (**b**) *η* = 0.84, (**c**) *η*_4_ = 0.8515, (**d**) *η* = 0.855, (**e**) *η*_5_ = 0.8589 and (**f**) *η* = 0.9. Note that in (**b**) the temperatures of the two cross points are denoted as *T*_1_ and *T*_2_, respectively.

The curves with larger *η* values are in Fig. 7. When *η* is reaches *η*_3_ = 0.825, the free energy curves of the two states tangent at an intermediate temperature point, see Fig. 7(a). Except at the tangency point, *F*_*AF*1_ is always less than *F*_*AF*2_. AF1 is more stable. Note that in this paper we only discuss the temperature range where *T* ≤ *T*_*N*_. As *η* increases from *η*_3_, the free energy curves of the two states begin to have two cross points, see Fig. 7(b), the temperatures of which are denoted as *T*_1_ and *T*_2_, respectively. At 0 ≤ *T* < *T*_1_ and *T*_2_ < *T* ≤ *T*_*N*_, *F*_*AF*1_ < *F*_*AF*2_, i.e., AF1 is more stable. At *T*_1_ < *T* < *T*_2_, *F*_*AF*1_ > *F*_*AF*2_, i.e., AF2 is more stable. At each cross point, an AF1-AF2 phase transformation may occur and it is a first-order phase transition.

When the value of *η* is up to *η*_4_ = 0.8515, we have *F*_*AF*1_(0^+^) = *F*_*AF*2_(0^+^) and the free energy curves of the two states touch at their ends, see Fig. 7(c). It is seen from Fig. 7(b),(c) that *η*_4_ is the upper limit where the two cross points appear. Therefore, as *η* > *η*_4_, the two cross points will disappear and there is only one cross left, see Fig. 7(d). This situation will last until *η*_5_ = 0.8589, see Fig. 7(e). Therefore, when *η*_4_ < *η* < *η*_5_, at temperature close to zero, *F*_*AF*1_(0^+^) > *F*_*AF*2_(0^+^), the AF2 is more stable. Very close to the critical temperature, *F*_*AF*1_(*T*) < *F*_*AF*2_(*T*), the AF1 is more stable. At the cross point, a first-order phase transition between AF1 and AF2 states may occur. At *η* = *η*_5_, the touch point of the two curves is just at the *T*_*N*_, *F*_*AF*1_(*T*) = *F*_*AF*2_(*T*), see Fig. 7(e). When *η* > *η*_5_, *F*_*AF*1_ is always greater than *F*_*AF*2_, so that AF2 is more stable, see Fig. 7(f).

**Figure 8:**
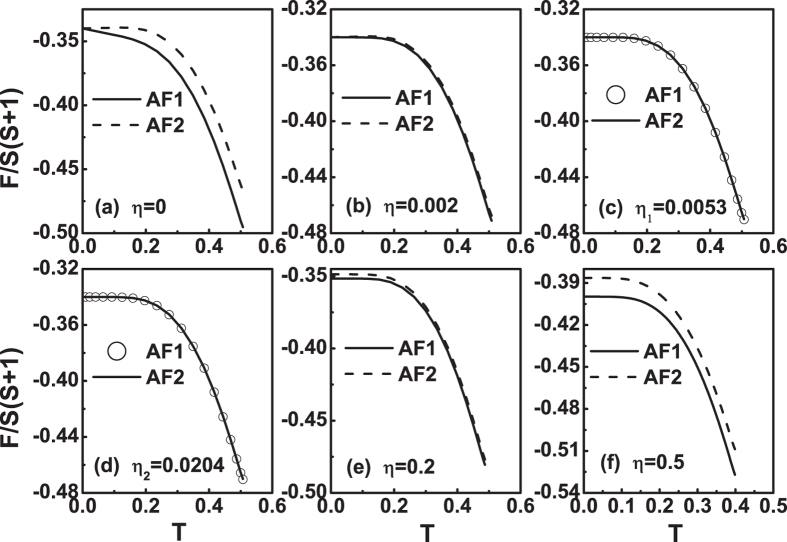
The free energy *F*(*T*) curves at *J*_3_ = −0.01 for different *η* values. (**a**) *η* = 0, (**b**) *η* = 0.002, (**c**) *η* = *η*_1_ = 0.0053, (**d**) *η* = *η*_2_ = 0.0204, (**e**) *η* = 0.2 and (**f**) *η* = 0.5.

**Figure 9:**
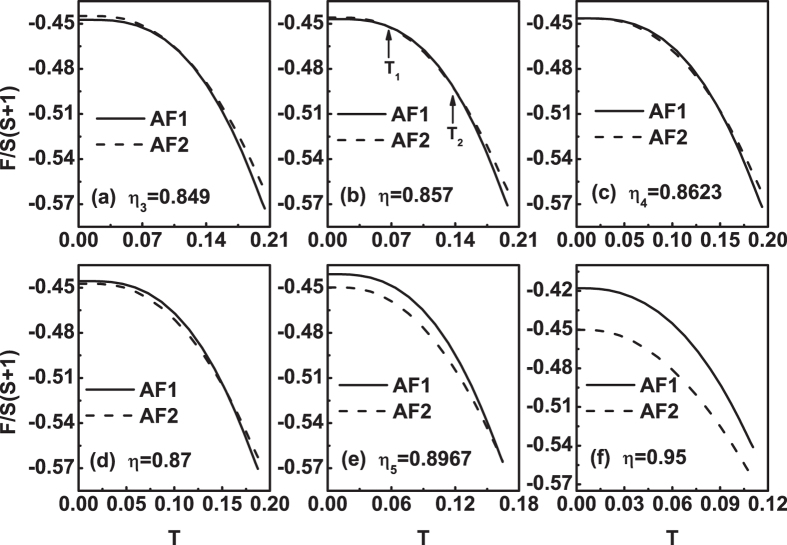
The free energy *F*(*T*) curves at *J*_3_ = − 0.01 for different *η* values. (**a**) *η*_3_ = 0.849, (**b**) *η* = 0.857, (**c**) *η*_4_ = 0.8623, (**d**) *η* = 0.87, (**e**) *η*_5_ = 0.8967 and (**f**) *η* = 0.95. Similar to Fig. 7(b), the temperatures of the two cross points in (**b**) are also denoted as *T*_1_ and *T*_2_, respectively.

Next, we discuss the case of *J*_3_ < 0. Figures 8 and 9 plot the free energy as a function of temperature for different *η* values when *J*_3_ = −0.01. Their results are similar to Figs 6 and 7. It is seen that *F*_*AF*1_ is always less than *F*_*AF*2_ when 0 ≤ *η* < *η*_1_ = 0.0053, see Fig. 8(a,b). When *η*_1_ ≤ *η* ≤ *η*_2_ = 0.0204, the difference between *F*_*AF*1_ and *F*_*AF*2_ is negligible, i.e., *F*_*AF*1_ = *F*_*AF*2_, see Fig. 8(c),(d). When *η*_2_ < *η* ≤ *η*_3_ = 0.0849, *F*_*AF*1_ is still less than *F*_*AF*2_ at *T* ≤ *T*_*N*_, see Fig. 9(a). As *η*_3_ < *η* < *η*_4_ = 0.8623, there are two cross points for the free energy curves of the two states, see Fig. 9(b),(c). Similar to Fig. 7(b),(c), at 0 ≤ *T* < *T*_1_ and *T*_2_ < *T* ≤ *T*_*N*_, *F*_*AF*1_ < *F*_*AF*2_, i.e., AF1 is more stable. At the temperature range in between, AF2 is more stable. At the two cross points, a first-order phase transition between AF1 and AF2 may occur. At *η* = *η*_4_, *F*_*AF*1_(0^+^) = *F*_*AF*2_(0^+^). As *η* increases from *η*_4_ to *η*_5_ = 0.8967, the two cross points will disappears and the free energy curves of the two states only one cross point left, see Fig. 9(d),(e). It indicates that at *η*_4_ < *η* < *η*_5_, *F*_*AF*1_(0^+^) > *F*_*AF*2_(0^+^), the AF2 is more stable. Near critical temperature, *F*_*AF*1_(*T*) < *F*_*AF*2_(*T*), so that the AF1 is more stable. For *η* = *η*_5_, at temperature close to *T*_*N*_, *F*_*AF*1_(*T*) = *F*_*AF*2_(*T*), see Fig. 9(e). When *η* > *η*_5_, *F*_*AF*1_ is always greater than *F*_*AF*2_, i.e., AF2 is more stable, see Fig. 9(f).

**Figure 10:**
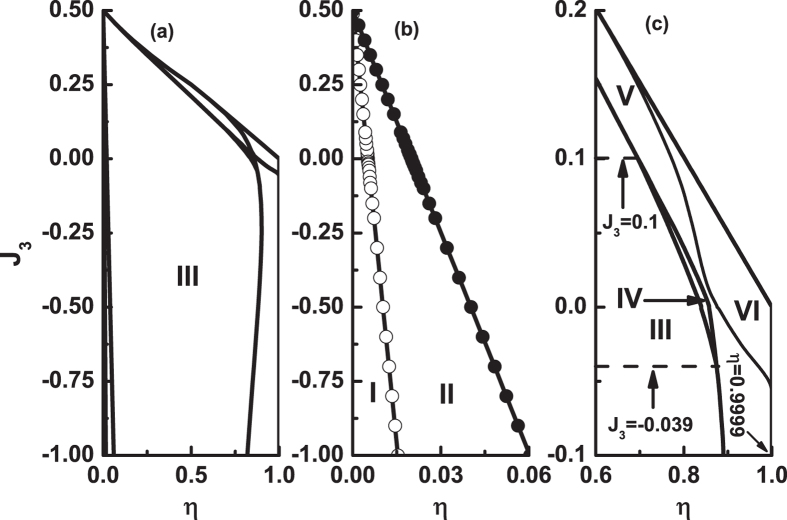
(**a**) The comparison of the free energies of the two states below the *T*_*N*_ in the *J*_3_ and *η* parameter space. There are six regions. (**b**) The enlargement of the region  − 1 ≤ *J*_3_ ≤ 0.5 and 0 ≤ *η* ≤ 0.06 in Fig. 10(a). (**c**) The enlargement of the region  −0.1 ≤ *J*_3_ ≤ 0.2 and 0.6 ≤ *η* < 1 in Fig. 10(a). The upper boundary line of region VI is just the line in Fig. 2(c). In regions I and III, *F*_*AF*1_ < *F*_*AF*2_. In region II, *F*_*AF*1_ = *F*_*AF*2_. In region IV, the free energy curves of the two states have two cross points. In region V, the free energy curves of the two states have one cross point. In region VI, *F*_*AF*1_ > *F*_*AF*2_.

We have seen from Figs 6–9 that at *J*_2_ = 0.5, there are various cases of the relationship, depending on the parameters *J*_3_ and *η*, between the free energies of the two configurations below the *T*_*N*_. All possible relationships are presented in Fig. 10(a). There are six regions in Fig. 10(a). Some of them are very narrow. Therefore, we illustrate in Fig. 10(b),(c) two enlargements. Figure 10(b) is the enlargement of the region −1 ≤ *J*_3_ ≤ 0.5 and 0 ≤ *η* ≤ 0.06 in Fig. 10(a), and Fig. 10(c) is the enlargement of the region − 0.1 ≤ *J*_3_ ≤ 0.2 and 0.6 ≤ *η* < 1 in Fig. 10(a). The six regions are marked by I to VI, respectively.

In region I, the free energy of AF1 is always less than that of AF2. The examples are the curves in Fig. 6(a),(b) and Fig. 8(a),(b). This region is denoted as *F*_*AF*1_ < *F*_*AF*2_.

In region II, the difference between the free energies of the two states is negligible. So it is denoted as *F*_*AF*1_ = *F*_*AF*2_. The examples are the curves in Fig. 6(c),(d) and Fig. 8(c),(d).

In region III, it is again that *F*_*AF*1_ < *F*_*AF*2_. The examples are the curves in Fig. 6(e),(f) and Fig. 8(e),(f).

In region IV, the *F*(*T*) curves of the two states have two cross points. The examples are the curves in Figs 7(b) and 9(b). The feature is that at temperatures close to zero, *F*_*AF*1_(0^+^) < *F*_*AF*2_(0^+^), in range of intermediate temperature, *F*_*AF*1_(*T*) > *F*_*AF*2_(*T*), and near the *T*_*N*_, *F*_*AF*1_(*T*) < *F*_*AF*2_(*T*). Therefore, as temperature is near zero, the state of the system should be AF1. As temperature rises, it is possible to occur a first-order phase transformation from the AF1 to AF2 at the first cross point, and then another first-order transition can happen from the AF2 to AF1 at the second cross point.

The region IV is rather narrow. Its *J*_3_ value range is between −0.039 and 0.1, as marked in Fig. 10(c), and its *η* value range is between 0.6955 and 0.8741.

In region V, the *F*(*T*) curves of the two states have one cross point. The examples are curves in Figs 7(d) and 9(d). The feature is that at temperatures close to zero, *F*_*AF*1_(0^+^) > *F*_*AF*2_(0^+^), and near the *T*_*N*_, *F*_*AF*1_(*T*) < *F*_*AF*2_(*T*). Therefore, as temperature is near zero, the state of the system should be AF2, and as temperature rises, it is possible to occur a first-order phase transformation from the AF2 to AF1 at the cross point below the *T*_*N*_.

In region VI, the free energy of AF1 is always greater than that of AF2, i.e., *F*_*AF*1_ > *F*_*AF*2_. The examples are the curves in Figs 7(f) and 9(f).

The boundary line between regions II and III can be expressed by *J*_3_ = − 25*η* + 0.5. The boundary line between regions I and II can be expressed by *J*_3_ = − 100*η* + 0.5. The upper boundary line of region VI is just the line in Fig. 2(c). It should be mentioned that in calculation, we take the *η* value up to *η* = 0.9999, as marked in Fig. 10(c).

### Possible phase transition at *J*_2_ ≠ *J*_1_/2

When *J*_2_ value is apart from 0.5, the two states can also coexist, as revealed by Figs 3 and 5. In these cases, the system should also be in the state with the lower free energy at any temperature. Then if the *F*(*T*) curves of the AF1 and AF2 states have cross points, there may occur phase transition between the two states.

**Figure 11:**
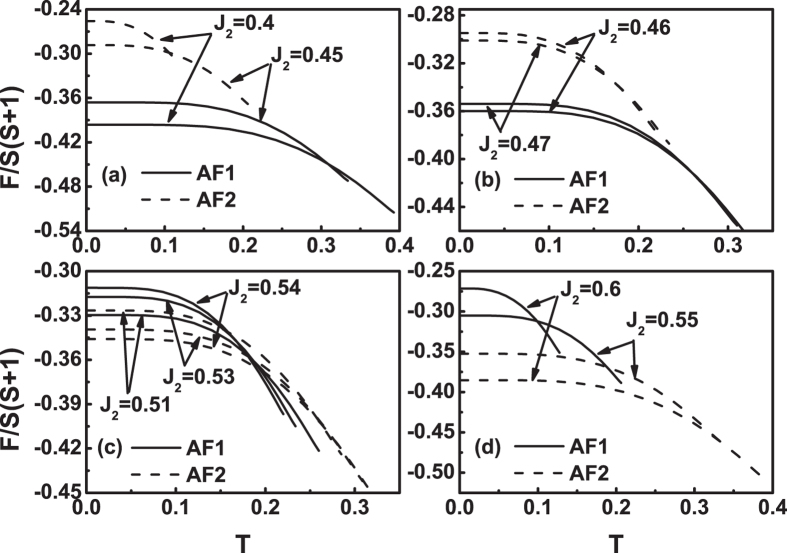
The free energy *F*(*T*) curves at *η* = 0.5 and *J*_3_ = 0.1 for various *J*_2_ values. (**a**) *J*_2_ = 0.4, 0.45, (**b**) *J*_2_ = 0.46, 0.47, (**c**) *J*_2_ = 0.51, 0.53, 0.54, (**d**) *J*_2_ = 0.55, 0.6.

Figure 11 plots the free energy as a function of temperature for different *J*_2_ values when *J*_3_ = 0.1 and *η* = 0.5. Two features are obvious in Fig. 11(a) to (d). One is that as *J*_2_ value increases, the *T*_*N*_ value of AF1 decreases and that of AF2 increases, which agrees with Fig. 3(a). The other is that on the whole, with the *J*_2_ value increasing, the *F*(*T*) curve of AF1 shifts upward and that of AF2 downward. As *J*_2_ = 0.4, the whole *F*(*T*) curve of AF2 is well above that of AF1, see Fig. 11(a), and so AF1 state is more stable. The *F*(*T*) curves of the AF1 and AF2 gradually become closer, see Fig. 11(b). At *J*_2_ = 0.5, the *T*_*N*_ values of both states are the same, as shown by Fig. 3(a), but the *F*(*T*) curve of the AF1 is still below that of AF2. At *J*_2_ = 0.5125, the free energies of the two states at zero temperature is negligible, i.e., *F*_*AF*1_(0^+^) = *F*_*AF*2_(0^+^). As *J*_2_ > 0.5125, *F*_*AF*1_(0^+^) > *F*_*AF*2_(0^+^), and the two curves have a cross below the *T*_*N*_, see Fig. 11(c). Thus, at temperature close to zero, the AF2 is more stable, and with temperature rising, there may occur a first-order transition from the AF2 to AF1 at the cross point. This case remains until *J*_2_ = 0.5658. As *J*_2_ > 0.5658, the whole *F*(*T*) curve of AF1 becomes well above that of AF2, see Fig. 11(d), and so AF2 state is always more stable.

**Figure 12:**
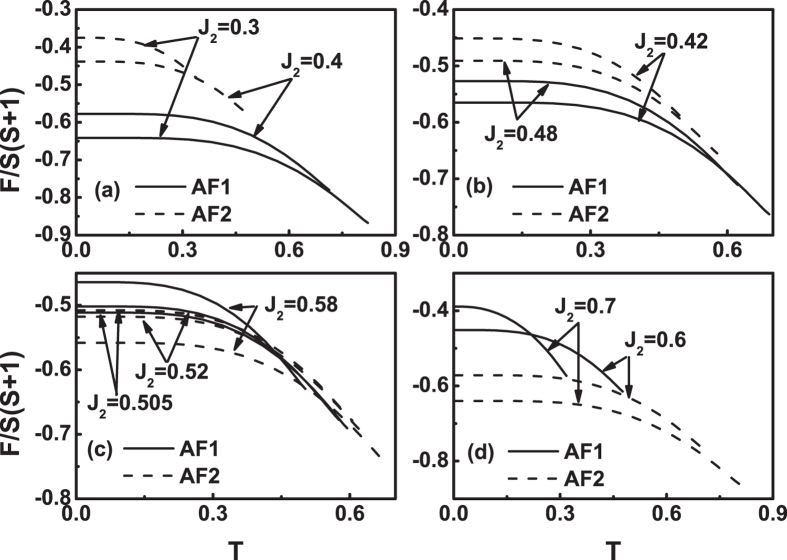
The free energy *F*(*T*) curves at *η* = 0.5 and *J*_3_ = −0.2 for various *J*_2_ values. (**a**) *J*_2_ = 0.3, 0.4, (**b**) *J*_2_ = 0.42, 0.48, (**c**) *J*_2_ = 0.505, 0.52, 0.58, (**d**) *J*_2_ = 0.6, 0.7.

The case of negative *J*_3_ value is illustrated in Fig. 12 where *J*_3_ = −0.1 and *η* = 0.5. The overall behavior in Fig. 12 is quite similar to that in Fig. 11. Two features are obvious in Fig. 12(a) to (d). One is that as *J*_2_ value increases, the *T*_*N*_ value of AF1 decreases and that of AF2 increases, which agrees with Fig. 5(a). The other is that on the whole, with the *J*_2_ value increasing, the *F*(*T*) curve of AF1 shifts upward and that of AF2 downward. As *J*_2_ = 0.3 to 0.48, the whole *F*(*T*) curve of AF2 is well above that of AF1, see Fig. 12(a) and (b), and so AF1 state is more stable. Meanwhile, the *F*(*T*) curves of the AF1 and AF2 gradually become closer. At *J*_2_ = 0.5, the *T*_*N*_ values of both states are the same, as shown by Fig. 5(a), but the *F*(*T*) curve of the AF1 is still below that of AF2. At *J*_2_ = 0.5078, the free energies of the two states at zero temperature is negligible, i.e., *F*_*AF*1_(0^+^) = *F*_*AF*2_(0^+^). As 0.5078 < *J*_2_ ≤ 0.5865, *F*_*AF*1_(0^+^) > *F*_*AF*2_(0^+^), and the two curves have a cross below the *T*_*N*_, see Fig. 12(c). Thus, at temperature close to zero, the AF2 is more stable, and with temperature rising, there may occur a first-order transition from the AF2 to AF1 at the cross point. As *J*_2_ > 0.5865, the whole *F*(*T*) curve of AF1 becomes well above that of AF2, see Fig. 12(d), and so AF2 state is more stable.

For the case of *J*_2_ ≠ *J*_1_/2, regardless of whether *J*_3_ is greater than or less than zero, we can obtain three conclusions. (1) The higher the *T*_*N*_, the lower the *F*_*AF*2_(0^+^), and the larger the difference between *T*_*N*_ s of AF1 and AF2 states, the larger the difference between *F*_*AF*1_(0^+^) and *F*_*AF*2_(0^+^). (2) For *J*_2_ <  0.5, *F*_*AF*1_(*T*) is always less than *F*_*AF*2_(*T*), i.e., in this case, AF1 is always more stable. (3) When *J*_2_ > 0.5, one should distinguish three cases. (1) When 0.5 < *J*_2_ < *J*_2,*c*1_, *F*_*AF*1_(*T*) is still less than *F*_*AF*2_(*T*). (2) When *J*_2,*c*1_ < *J*_2_ < *J*_2,*c*2_, *F*_*AF*1_(0^+^) becomes greater than *F*_*AF*2_(0^+^), and the free energy curves of the two states have a cross point, at which a first-order phase transition between AF1 and AF2 states may occur. (3) When *J*_2_ > *J*_2,*c*2_, *F*_*AF*1_(*T*) is always greater than *F*_*AF*2_(*T*). The *J*_2,*c*1_ and *J*_2,*c*2_ values depend on *J*_3_ value.

## Discussions

In this paper, by means of the double-time Green’s function method, the finite-temperature magnetic properties of the frustrated spin-1/2 *J*_1_-*J*_2_-*J*_3_ antiferromagnet on the 2D square lattice have been investigated under random phase approximation. Our results have shown for the case of *J*_3_ > 0 that the Néel state and collinear state have the same critical temperature at *J*_2_ = *J*_1_/2 when the *J*_3_ and *η* take value in the range of $\mathrm{0 < }{J}_{3}\le {J}_{3}^{c}$ and 0 ≤ *η* ≤ *η* ^*c*^. Beyond this range, it is a paramagnetic phase at *J*_2_ = *J*_1_/2. For *J*_3_ < 0, under the condition of 0 ≤ *η* < 1, the critical temperature of AF1 is always equal to AF2 as long as *J*_2_ = *J*_1_/2. For the case of *J*_2_ ≠ *J*_1_/2, our results indicate that both states can exist, while they have different critical temperatures. Thus a possible phase transition between the Néel state and collinear state with the case of *J*_2_ = *J*_1_/2 and *J*_2_ ≠ *J*_1_/2 has been also discussed, respectively.

In order to discuss explicitly which state is more stable, free energy as a function of temperature is calculated.

For *J*_2_ = *J*_1_/2, our results show that there are six cases of the relationship between the free energies of the AF1 and AF2 states depending on the *J*_3_ and *η* values, see Fig. 10. In regions I and III, *F*_*AF*1_(*T*) < *F*_*AF*2_(*T*), AF1 state is more stable. In region II, the difference between the free energies of the AF1 and AF2 is negligible, i.e., *F*_*AF*1_(*T*) = *F*_*AF*2_(*T*). In this case, the system can be in either the AF1 or AF2 state or a coexistence of them. In region IV, the two free energy curves have two cross points. In the temperature range between the two cross points, AF2 is more stable, while outside of this range, AF1 is more stable. In region V, the two free energy curves have one cross point. At temperature close to zero, AF2 is more stable, and above the cross point, AF1 is more stable. In region VI, *F*_*AF*1_(*T*) > *F*_*AF*2_(*T*), AF2 state is more stable.

For the case of *J*_2_ ≠ *J*_1_/2, when *J*_2_ < *J*_1_/2, the AF1 is always more stable than AF2 below *T*_*N*_. But for *J*_2_ > *J*_1_/2, there are three cases. (1) When *J*_2_ takes value in the vicinity of *J*_1_/2, the AF1 is more stable. (2) When the *J*_2_ value increases further, a first-order phase transition between these two states may occur. Therefore, in this case, when temperature approaches zero temperature, the AF2 is more stable. Near the critical point, the AF1 state is more stable. (3) When the *J*_2_ value continues increase, the AF2 is more stable.

## Model and Method

The Hamiltonian of *J*_1_-*J*_2_-*J*_3_ model can be written as

1 $$\begin{array}{rcl}H & = & {J}_{1}\sum _{ < i,j > }[\frac{\eta }{2}({S}_{i}^{x}{S}_{j}^{x}+{S}_{i}^{y}{S}_{j}^{y})+{S}_{i}^{z}{S}_{j}^{z}]+{J}_{2}\sum _{ <  < i,j >  > }[\frac{\eta }{2}({S}_{i}^{x}{S}_{j}^{x}+{S}_{i}^{y}{S}_{j}^{y})+{S}_{i}^{z}{S}_{j}^{z}]\\  &  & +{J}_{3}\sum _{[i,j]}[\frac{\eta }{2}({S}_{i}^{x}{S}_{j}^{x}+{S}_{i}^{y}{S}_{j}^{y})+{S}_{i}^{z}{S}_{j}^{z}]\mathrm{.}\end{array}$$

where ${S}_{i}^{x}$, ${S}_{i}^{y}$ and ${S}_{i}^{z}$ represent the three components of the spin-*S* operator for a spin at site *i*. The denotations <*i*, *j*>, ≪*i*, *j*≫ and [*i*, *j*] mean the summations over the NN, NNN and NNNN lattice sites, respectively. The symbol *η* denotes the anisotropic parameters with 0 ≤ *η* < 1. Spin quantum number is *S* = 1/2 and the lattice is the 2D square one. In this paper, we set *J*_1_ = 1 and *J*_2_ > 0.

For the sake of convenience, we let Boltzmann constant *k*_*B*_ = 1 so that all the quantities, including Hamiltonian parameters, temperature *T*, and sublattice magnetization *m* = 〈*S* ^*z*^〉, become dimensionless. 〈*S* ^*z*^〉 is the assembly statistical average of spin operator *S* ^*z*^.

We use the DTGF method and introduce the following Greens function (GF)^42^

2 $${G}_{ij}^{\pm }=\langle \langle {S}_{i}^{\pm };{e}^{u{S}_{j}^{z}}{S}_{j}^{-}\rangle \rangle \mathrm{.}$$

Here, *u* is a parameter^40^. After solving the Green’s function by means of the method of equation of motion, *u* will be ultimately set as zero to give the expression of magnetization^40^. We derive the equation of motion of the Green’s function by a standard procedure^40, 42^. In the course of derivation, the higher order Green functions appearing in the equation of motion have to be decoupled. In this paper, we apply random phase approximation (RPA)^40, 42^ to decouple the higher order GFs,

3 $$\langle \langle {S}_{l}^{z}{S}_{i}^{\pm };{e}^{u{S}_{j}^{z}}{S}_{j}^{-}\rangle \rangle =\langle {S}_{l}^{z}\rangle \langle \langle {S}_{i}^{\pm };{e}^{u{S}_{j}^{z}}{S}_{j}^{-}\rangle \rangle ;l\ne i\mathrm{.}$$

After decoupling the higher order GFs and standard procedure^40, 42^, we obtain

4 $$\frac{2}{N}\sum _{k}\langle {e}^{u{S}_{i}^{z}}{S}_{i}^{-}{S}_{i}^{+}\rangle (k)=\theta (u){\phi }_{F},\,\,F=AF1,AF\mathrm{2,}$$

where the summation of wave vector *k* runs over the first Brillouin zone. *N* is the number of lattice sites and

5 $$\theta (u)=\langle [{S}_{i}^{+},{e}^{u{S}_{j}^{z}}{S}_{j}^{-}]\rangle \mathrm{.}$$

For *u* = 0, *θ*(*u*) = 2*m*. Using Eqs (4) and (5), we obtain

6 $${\varphi }_{F}=\frac{2}{N}\sum _{k}\frac{{E}_{1F}}{2\sqrt{{E}_{1F}^{2}-{E}_{2F}^{2}}}\,\coth \,\frac{\sqrt{{E}_{1F}^{2}-{E}_{2F}^{2}}}{2T}-\frac{1}{2}\mathrm{.}$$

Here for the AF1 state,

7 $$\begin{array}{rcl}{E}_{1AF1} & = & 2m\mathrm{[2}{J}_{1}+2{J}_{2}(\eta {\gamma }_{2k}-\mathrm{1)}+{J}_{3}(\eta {\gamma }_{3k}-\mathrm{2)],}\\ {E}_{2AF1} & = & 2{J}_{1}m\eta {\gamma }_{1k},\end{array}$$

and for the AF2 state,

8 $$\begin{array}{rcl}{E}_{1AF2} & = & 2m[{J}_{1}\eta {\gamma }_{1ak}+2{J}_{2}+2{J}_{3}(\eta {\gamma }_{3k}-\mathrm{2)],}\\ {E}_{2AF2} & = & 2\eta m({J}_{1}{\gamma }_{1bk}+2{J}_{2}{\gamma }_{2k}\mathrm{).}\end{array}$$

where

9 $$\begin{array}{rcl}{\gamma }_{1ak} & = & \cos \,{k}_{x},{\gamma }_{1bk}=\,\cos \,{k}_{y},{\gamma }_{1k}={\gamma }_{1ak}+{\gamma }_{1bk},\\ {\gamma }_{2k} & = & \cos \,{k}_{x}\,\cos \,{k}_{y},{\gamma }_{3k}=\,\cos \,2{k}_{x}+\,\cos \,2{k}_{y}\mathrm{.}\end{array}$$

Using Eqs (5), (6) and the relation $\langle {S}_{i}^{-}{S}_{i}^{+}\rangle =S(S+\mathrm{1)}-\langle {S}_{i}^{z}\rangle -\langle {({S}_{i}^{z})}^{2}\rangle$, the sublattice magnetization is expressed by following formula^40, 42^

10 $$m=\frac{({\varphi }_{F}+1+S){\varphi }_{F}^{2S+1}-({\phi }_{F}-S)({\varphi }_{F}+{\mathrm{1)}}^{2S+1}}{{({\varphi }_{F}+\mathrm{1)}}^{2S+1}-{\varphi }_{F}^{2S+1}}\mathrm{.}$$

The Néel points of the two configurations are expressed by

11 $$\frac{{k}_{B}{T}_{N,AF1}}{S(S+\mathrm{1)}}=\frac{m}{3}(\frac{2}{N}\sum _{k}\frac{{E}_{1AF1}}{{E}_{1AF1}^{2}-{E}_{2AF1}^{2}}{)}^{-1},$$

and

12 $$\frac{{k}_{B}{T}_{N,AF2}}{S(S+\mathrm{1)}}=\frac{m}{3}{(\frac{2}{N}\sum _{k}\frac{{E}_{1AF2}}{{E}_{1AF2}^{2}-{E}_{2AF2}^{2}})}^{-1},$$

respectively.

## References

[1] Misguich, G. & Lhuillier, C. in *Frustrated Spin Systems* (2nd edn, edited by Diep, H. T.) 235–304 (Singapore, 2013).

[2] Viana JR, deSousa JR (2007). Anisotropy effects in frustrated Heisenberg antiferromagnets on a square lattice. Phys. Rev. B.

[3] Ren YZ, Tong NH, Xie XC (2014). Cluster mean-field theory study of *J*_1_-*J*_2_ Heisenberg model on a square lattice. J. Phys.: Condens. Matter.

[4] Gong SS, Zhu W, Sheng DN, Motrunich OI, Fisher MPA (2014). Plaquette Ordered Phase and Quantum Phase Diagram in the Spin-1/2Square Heisenberg Model. Phys. Rev. Lett..

[5] Jiang HC, Yao H, Balents L (2012). Spin liquid ground state of the spin-1/2 *J*_1_-*J*_2_ Heisenberg model. Phys. Rev. B.

[6] Schmidt B, Siahatgar M, Thalmeier P (2011). Ordered moment in the anisotropic and frustrated square lattice Heisenberg model. Phys. Rev. B.

[7] Darradi R (2008). Ground state phases of the spin-1/2 *J*_1_-*J*_2_ Heisenberg antiferromagnet on the square lattice: A high-order coupled cluster treatment. Phys. Rev. B.

[8] Doretto RL (2014). Plaquette valence-bond solid in the square-lattice *J*_1_-*J*_2_ antiferromagnet Heisenberg model: A bond operator approach. Phys. Rev. B.

[9] Roscilde T, Feiguin A, Chernyshev AL, Liu S, Haas S (2004). Anisotropy-Induced Ordering in the Quantum-1/2 Antiferromagnet. Phys. Rev. Lett..

[10] Majumdar K (2010). Second-order quantum corrections for the frustrated spatially anisotropic spin-1/2 Heisenberg antiferromagnet on a square lattice. Phys. Rev. B.

[11] Bishop RF, Li PHY, Farnell DJJ, Campbell CE (2009). Magnetic order in a spin-1/2 interpolating square-triangle Heisenberg antiferromagnet. Phys. Rev.B.

[12] Bishop RF, Li PHY, Darradi R, Schulenburg J, Richter J (2008). Effect of anisotropy on the ground-state magnetic ordering of the spin-half quantum *J*_1_^*XYZ*^–*J*_2_^*XYZ*^ model on the square lattice. Phys. Rev. B.

[13] Zhang YY (2009). Localization and the Kosterlitz-Thouless Transition in Disordered Graphene. Phys. Rev. Lett..

[14] Ji AC, Liu WM, Song JL, Zhou F (2008). Dynamical Creation of Fractionalized Vortices and Vortex Lattices. Phys. Rev. Lett..

[15] Ji AC, Xie XC, Liu WM (2007). Quantum Magnetic Dynamics of Polarized Light in Arrays of Microcavities. Phys. Rev. Lett..

[16] Iqbal Y, Hu WJ, Thomale R, Poilblanc D, Becca F (2016). Spin liquid nature in the Heisenberg *J*_1_-*J*_2_ triangular antiferromagnet. Phys. Rev. B.

[17] Hu WJ, Gong SS, Zhu W, Sheng DN (2015). Competing spin-liquid states in the spin-1/2 Heisenberg model on the triangular lattice. Phys. Rev. B.

[18] Zhu ZY, W SR (2015). Spin liquid phase of the S = 1/2 *J*_1_-*J*_2_ Heisenberg model on the triangular lattice. Phys. Rev. B.

[19] Zhang GM, Hu H, Yu L (2003). Valence-Bond Spin-Liquid State in Two-Dimensional Frustrated Spin-1/2 Heisenberg Antiferromagnets. Phys. Rev. Lett..

[20] Li PHY, Bishop RF (2016). Ground-state phases of the spin-1 *J*_1_-*J*_2_ Heisenberg antiferromagnet on the honeycomb lattice. Phys. Rev. B.

[21] Saadatmand SN, McCulloch IP (2016). Symmetry fractionalization in the topological phase of the spin-1/2 *J*_1_-*J*_2_ triangular Heisenberg model. Phys. Rev. B.

[22] Gong SS, Zhu W, Sheng DN (2015). Quantum phase diagram of the spin-1/2 Heisenberg model on the honeycomb lattice. Phys. Rev. B.

[23] Capponi S, Derzhko O, Honecker A, Läuchli AM, Richter J (2013). Numerical study of magnetization plateaus in the spin-1/2 kagome Heisenberg antiferromagnet. Phys. Rev. B.

[24] Rousochatzakis I, Moessner R, Brink Jvd (2013). Frustrated magnetism and resonating valence bond physics in two-dimensional kagome-like magnets. Phys. Rev. B.

[25] Richter J, Derzhko O, Schulenburg J (2004). Magnetic-Field Induced Spin-Peierls Instability in Strongly Frustrated Quantum Spin Lattices. Phys. Rev. Lett..

[26] Coldea R (2001). Spin Waves and Electronic Interactions in La_2_CuO_4_. Phys. Rev. Lett..

[27] Carretta P (2002). Frustration-driven structural distortion in VOMoO_4_. Phys. Rev. B.

[28] Melzi R (2000). Li_2_VO(Si, Ge)O_4_, a Prototype of a Two-Dimensional Frustrated Quantum Heisenberg Antiferromagnet. Phys. Rev. Lett..

[29] Pavarini E (2008). Effect of high pressure on competing exchange couplings in Li_2_VOSiO_4_. Phys. Rev. B.

[30] Mermin ND, Wagner H (1966). Absence of Ferromagnetism or Antiferromagnetism in One- or Two-Dimensional Isotropic Heisenberg Models. Phys. Rev. Lett..

[31] Liu RM (2016). Role of further-neighbor interactions in modulating the critical behavior of the Ising model with frustration. Phys. Rev. E.

[32] Yu R, Si QM (2015). Antiferroquadrupolar and Ising-Nematic Orders of a Frustrated Bilinear-Biquadratic Heisenberg Model and Implications for the Magnetism of FeSe. Phys. Rev. Lett..

[33] Ma FJ, Ji W, Hu JP, Lu ZY, Xiang T (2009). First-Principles Calculations of the Electronic Structure of Tetragonal α-FeTe and α-FeSe Crystals: Evidence for a Bicollinear Antiferromagnetic Order. Phys. Rev. Lett..

[34] Moreo A, Dagotto E, Jolicoeur T, Riera J (1990). Incommensurate correlations in the *t*-*j* and frustrated spin-1/2 Heisenberg models. Phys. Rev. B.

[35] Chubukov A (1991). First-order transition in frustrated quantum antiferromagnets. Phys. Rev. B.

[36] Ferrer J (1993). Spin-liquid phase for the frustrated quantum Heisenberg antiferromagnet on a square lattice. Phys. Rev. B.

[37] Mambrini M, Läuchli A, Poilblanc D, Mila F (2006). Plaquette valence-bond crystal in the frustrated Heisenberg quantum antiferromagnet on the square lattice. Phys. Rev. B.

[38] Reuther J, Abanin DA, Thomale R (2011). Magnetic order and paramagnetic phases in the quantum *J*_1_-*J*_2_ honeycomb model. Phys. Rev. B.

[39] Capriotti L, Sachdev S (2004). Low-Temperature Broken-Symmetry Phases of Spiral Antiferromagnets. Phys. Rev. Lett..

[40] Fröbrich P, Kuntz PJ (2006). Many-body Green’s function theory of Heisenberg films. Phys. Rep..

[41] Wang HY, Zhai LJ, Qian MC (2014). The internal energies of Heisenberg magnetic systems. J. Magn. Magn. Mater..

[42] Wang, H. Y. *Green’s Function in Condensed Matter Physics* 348-374 (Beijing, 2012).

